# The Clinical Validation of a Common Analytical Change Criteria for Cardiac Troponin for Ruling in an Acute Cardiovascular Outcome in Patients Presenting with Ischemic Chest Pain Symptoms

**DOI:** 10.3390/jcdd10080335

**Published:** 2023-08-04

**Authors:** Peter A. Kavsak, Sameer Sharif, Isabella Globe, Craig Ainsworth, Jinhui Ma, Matthew McQueen, Shamir Mehta, Dennis T. Ko, Andrew Worster

**Affiliations:** 1 Department of Pathology and Molecular Medicine, McMaster University, Hamilton, ON L8S 4L8, Canada; 2 Department of Medicine, McMaster University, Hamilton, ON L8S 4L8, Canada; 3 Faculty of Arts and Science, Queen’s University, Kingston, ON K7L 3N6, Canada; 4 Department of Health Research Methods, Evidence, and Impact, McMaster University, Hamilton, ON L8S 4L8, Canada; 5 Sunnybrook Hospital, Toronto, ON M4N 3M5, Canada

**Keywords:** high-sensitivity cardiac troponin, change criteria, myocardial infarction, acute coronary syndrome, emergency department

## Abstract

Serial cardiac troponin (cTn) testing on patients with symptoms suggestive of acute coronary syndrome (ACS) is primarily to identify those patients with evolving myocardial injury. With the improved analytical performance of the high-sensitivity cTn (hs-cTn) assays, different change criteria have been proposed that are mostly assay dependent. Here, we developed and compared a new Common Change Criteria (3C for the combined criteria of >3 ng/L, >30%, or >15% based on the initial cTn concentration of <10 ng/L, 10 to 100 ng/L, or >100 ng/L, respectively) method, versus the 2 h assay-dependent absolute change criteria endorsed by the European Society of Cardiology (ESC), versus the common relative >20% change criterion. These different analytical change criteria were evaluated in 855 emergency department (ED) patients with symptoms of ACS and who had two samples collected 3 h apart. The cTn concentrations were measured with four different assays (Abbott hs-cTnI, Roche hs-cTnT, Ortho cTnI-ES, and Ortho hs-cTnI). The outcomes evaluated were myocardial infarction (MI) and a composite outcome (MI, unstable angina, ventricular arrhythmia, heart failure, or cardiovascular death) within 7 days of ED presentation. The combined change criteria (3C) method yielded higher specificities (range: 93.9 to 97.2%) as compared to the >20% criterion (range: 42.3 to 88.1%) for all four assays for MI. The 3C method only yielded a higher specificity estimate for MI for the cTnI-ES assay (95.9%) versus the absolute change criteria (71.7%). Similar estimates were obtained for the composite outcome. There was also substantial agreement between hs-cTnT and the different cTnI assays for MI with the 3C method, with the percent agreement being ≥95%. The Common Change Criteria (3C) method combining both absolute and different percent changes may be used with cTnI, hs-cTnT, and different hs-cTnI assays to yield similar high-specificity (rule-in) estimates for adverse cardiovascular events for patients presenting to the ED with ACS symptoms.

## 1. Introduction

For over 40 years, guidelines for the interpretation of laboratory biomarkers in the diagnosis of myocardial infarction (MI) have emphasized a change in biomarker concentrations with serial measurements [1,2,3,4,5,6]. With the redefinition of MI in 2000, the emphasis was placed on cardiac troponin (cTn), with serial determinations important to help interpret concentrations near the upper reference limit (URL being the 99th percentile) [7]. The 2007 Universal Definition of MI recommended a relative (20%) change in cTn concentrations should be considered significant at higher concentrations where the coefficient of variation (CV) is approximately 5–7% [3]. Since 2007, analytical improvements in cTn precision and sensitivity have produced a newer, preferred generation of assays referred to as high-sensitivity cardiac troponin (hs-cTn) [5,6,8,9].

The early clinical validation of the hs-cTn assays assessed change using relative (%) or absolute cTn concentration changes, with early findings suggesting superior performance for the absolute change criteria [10,11,12]. The latest European Society of Cardiology (ESC) recommendations have provided assay-specific change criteria that can be used to rule-in MI, with the 4th Universal Definition of MI also stating a change of >20% in cTn concentrations being important for Type 4 and 5 MIs, with a cTn stable level being ≤20% variation, which is important in order to distinguish chronic from acute injury [5,6].

Recently, common analytical criteria have been published for hs-cTn assays; specifically, allowable variation would be ± 3 ng/L for concentrations <10 ng/L and ±30% for concentrations slightly above and around the 99th percentiles [13]. At higher concentrations, there has been less emphasis on what is the acceptable analytical variation, with a recent long-term publication (data over 7 years) indicating a 15% cutoff would be suitable [14]. The objective of the present study was to evaluate cTn change alone for ruling-in an MI or a composite acute cardiovascular event across four different cTn assays using the 20% criterion, the ESC absolute cutoffs, and a new Common Change Criteria (3C) in patients presenting to the emergency department (ED) with symptoms suggestive of acute coronary syndrome (ACS). 

## 2. Methods

### 2.1. Study Cohort

The study population has been previously described (Clinicaltrials.gov: NCT01994577) [15,16,17,18]. In brief, after obtaining Research Ethics Board (REB) approval, adult patients (18 years and older) who arrived at the ED (not transferred from another hospital) and had cTn ordered (Abbott ARCHITECT cTnI assay) by the ED physician were screened for study entry. Participants were excluded if they had an ST elevation MI at presentation, if their symptoms were not deemed to be due to ACS, had a previous MI or cardiac procedure/surgery or pulmonary embolus within 30 days, or history of malignancy or non-cardiac fatal disease or sepsis or ventricular fibrillation or sustained ventricular tachycardia at presentation. For this cohort, all participants had a least 2 samples (ED presentation and 3 h later) measured by 4 different assays (Abbott hs-cTnI, Roche hs-cTnT, Ortho cTnI-ES, Ortho hs-cTnI). The EDTA plasma samples were tested for the Abbott hs-cTnI and Roche hs-cTnT assays on fresh, not frozen samples [16]. An aliquot was frozen (below −70 °C) and thawed for the first time and tested for the Ortho cTnI assays, with data supporting stability after multiple freeze–thaw cycles and over 15 years when frozen below −70 °C [17,19]. The treating clinicians and the clinical adjudication team were blinded to the results of these 4 cTn assays [15,16,17,18]. It is important to note that the Ortho ES cTnI assay is not a high-sensitivity assay [17,20].

### 2.2. Outcomes

The 3rd Universal Definition of MI was used for the diagnosis of MI [4]. Clinically, cTnI (Abbott ARCHITECT) was reported in these patients with a cTnI concentration >99th percentile (0.03 µg/L) used to identify myocardial injury with an absolute change in concentration being ≥0.03 µg/L for concentrations <0.1 µg/L and a relative change in concentration ≥20% for concentrations ≥0.1 µg/L, or new ST segment elevation or depression indicative of ischemia, new left bundle branch block, coronary artery intervention, or pathologic findings of acute MI [15]. For the composite outcome, in addition to MI, unstable angina, heart failure, serious ventricular arrhythmia, and cardiovascular death within 7 days of ED presentation were included, as previously described [15]. A team independently adjudicated the outcomes, and in the event that consensus between two reviewers was not possible, a third reviewer was used. There was no distinction made for the type of MI, with the adjudicators being blinded to the Abbott hs-cTnI, Roche hs-cTnT, Ortho cTnI-ES, and Ortho hs-cTnI levels. 

### 2.3. Statistical Analysis

Change in cTn concentrations were assessed as follows. For concentrations >20%, the difference between the 2nd from the 1st sample was divided by the 1st sample concentration, with an absolute percent >20% (i.e., rise or fall) being designated as positive for change. For the ESC absolute change, the published criteria for rule in was used based on the 2 h algorithm pathways with the absolute difference between the 2nd and 1st sample used to detect change: Abbott hs-cTnI ≥15 ng/L (or >14 ng/L) and Roche hs-cTnT ≥10 ng/L (or >9 ng/L) [6]. The Ortho hs-cTnI 2 h absolute change criterion was published after the ESC recommendations and is listed as ≥5 ng/L (or >4 ng/L) [21,22]. The same absolute change criterion was used for the Ortho cTnI-ES assay, and prior to calculating change, the Ortho cTnI-ES concentrations in µg/L were multiplied by 1000 to yield ng/L values [17]. 

The new common change criteria or 3C utilized combined absolute and relative change criteria of >3 ng/L, >30%, or >15% based on the initial cTn concentration of <10 ng/L, 10 to 100 ng/L, or >100 ng/L, respectively. Briefly, if the 1st sample had a cTn concentration <10 ng/L, then a change would be detected if the difference between the 2nd from the 1st was >3 ng/L (absolute level) [13]. If the 1st sample had a cTn concentration between 10 ng/L and 100 ng/L, then a difference >30% (absolute percent) was needed to detect a change [13]. If either the first or second sample had a concentration >100 ng/L, then a difference >15% (absolute percent) was needed to detect change [14]. The 100 ng/L is equivalent to 0.1 µg/L with the non-hs-cTn assays where a change of 20% was commonly used to detect change [23]. The 3C method was derived from analytical imprecision and accuracy estimates from laboratories that were and are involved in the CODE-MI (NCT03819894) and VISION Cardiac Surgery (NCT01842568) studies [13,14]. Descriptive analyses were performed with diagnostic estimates (specificity, positive predictive value (PPV), positive likelihood ratio (PLR), sensitivity, negative predictive value, and negative likelihood ratio) and kappa for agreement with 95% confidence intervals (CI) provided. For the kappa value calculation, each of the 3 change criteria was compared between the different cTnI assays and to hs-cTnT (i.e., change present and change absent for each cTnI assay compared to hs-cTnT). Each of the assays and change criteria was then assessed against the published benchmarks of a specificity ≥ 90%, PPV ≥ 75%, and a PLR ≥ 10 [24,25]. We performed all analyses using MedCalc for Windows, version 22.006 (MedCalc Software, Ostend, Belgium) and Graphpad prism software (QuickCalcs Web site: http://www.graphpad.com/quickcalcs/ConfInterval1.cfm accessed 30 June 2023). 

## 3. Results

**Figure 1:**
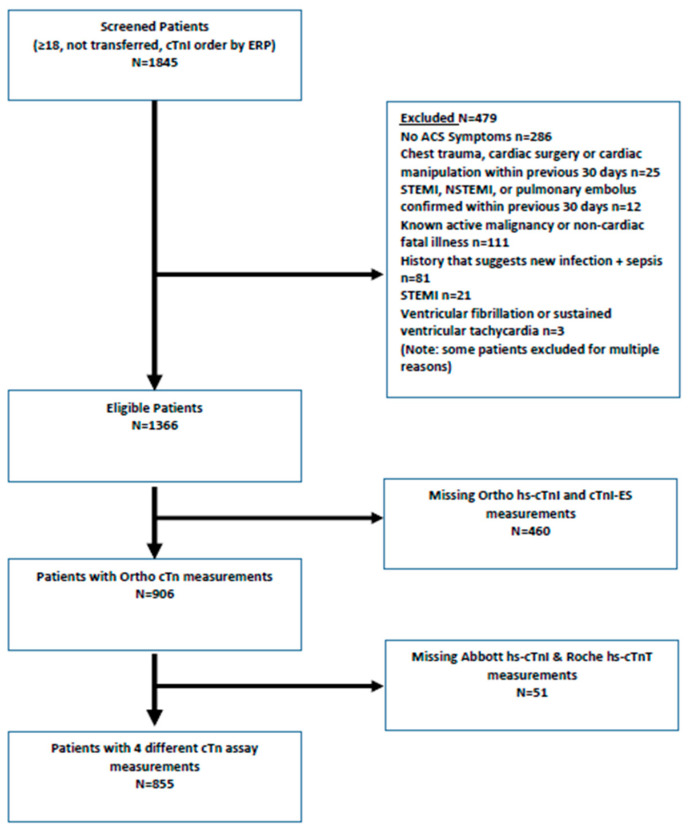
Flow diagram of study cohort. Abbreviations: ACS, acute coronary syndrome; STEMI, ST segment elevation myocardial infarction; NSTEMI, non-STEMI; cTnI, cardiac troponin I; hs-cTnI, high-sensitivity cardiac troponin I; hs-cTnT, high-sensitivity cardiac troponin T.

The cohort (*n* = 855), 53% of whom were female, that had all four cTn assays measured on both samples, had a median (interquartile) age of 68 years (56 to 80) (Figure 1). The Abbott hs-cTnI assay absolute change criterion (>14 ng/L) achieved a specificity of 98.6% (95% CI: 97.4 to 99.3%), a PPV of 83.1% (95% CI: 72.8 to 90.0%), and PLR of 46.9 (95% CI: 25.6 to 86.0) for MI (Table 1). No other assay nor change criteria exceeded the benchmarks for all three diagnostic parameters for MI. The highest observed specificity estimate when using the >20% criterion was with the Roche hs-cTnT assay for MI (specificity = 88.1%; 95% CI: 85.6 to 90.3%) (Table 2). The 3C method yielded high specificities in the range of 93.9% (Abbott hs-cTnI) to 97.2% (Roche hs-cTnT), with exactly the same estimates for Ortho cTnI-ES and Ortho hs-cTnI for MI (95.9%; 95% CI: 94.2 to 97.2%) (Table 3 and Table 4). 

For the composite outcome, the specificities for the 3C method were ≥95%, with the Ortho cTnI-ES assay diagnostic estimates exceeding the benchmarks for specificity (97.5%), PPV (76.4%), and PLR (11.6) (Table 3). The concordance between hs-cTnT and the other cTnI assays was substantial when the 3C method for change was used (i.e., 95% observed agreements) for MI (Table 5). The >20% criterion yielded the lowest kappa and percent agreements, with the highest observed PPV being 37.2% and PLR being 3.8 with the Roche hs-cTnT assay.

## 4. Discussion

The major finding of this study is the poor specificity for both MI and the composite outcome when using only the >20% change criterion. This contrasts with the newly derived Common Change Criteria (3C), where the specificities are similar across all assays and substantially higher (all >90%) for MI and the composite cardiovascular adverse events outcome. Similar performances can be achieved when using the hs-cTn assays with the ESC absolute change criteria, which are assay dependent [6]. Here, the Ortho cTnI-ES assay (which is not a hs-cTn assay) achieves similar performance to the hs-cTn assays only when the 3C method to identify change is used. The findings from this study indicate that the common analytical change criteria may be applicable across all cTn assays. Additional strengths of our study are the prospective sample collection, health outcomes assessed, and measurements with four different cTn assays that have different analytical characteristics and interferences [26,27,28].

A few limitations are notable. First, there is a reasonable analytical and clinical agreement between the Abbott hs-cTnI assay and Abbott cTnI assay that was used clinically [11,29]. This, in part, could explain the high diagnostic performance when using the absolute change criterion for the Abbott hs-cTnI assay. Second, the ESC algorithms and other pathways also incorporate a baseline value into the decision to rule-in MI in a patient [6,30]. The baseline level was used in the 3C method to select the appropriate change criterion and was not used alone to identify high-risk individuals. Third, the absolute cutoffs were used from the ESC 0/2 h algorithms, and our samples were collected 3 h apart. However, previous literature has suggested that early change criteria (i.e., 0/1 h or 0/2 h) can be applied to later sample draws (i.e., 0/3 h) with minimal impact on performance [18,31]. However, our study design would fall into the “late resampling” category with blood draws >120 to 210 min apart, and the performance for an “early resampling” (>45 to 120 min) protocol [30] using the 3C method would need to be evaluated. Fourth, longer-term cardiovascular outcomes using the 3C method were not assessed, and future studies should also evaluate if this change criteria can be used in convalescent settings following ACS for additional risk stratification [32,33]. Fifth, it is important to reiterate that the >20% criterion is recommended to distinguish acute from chronic myocardial injury, and the data from this study indicate that the application of this percent change across all concentration levels leads to lower specificity. However, the analytical change used in the 3C method finds support from recent studies assessing reference change values (RCV) for hs-cTn assays, where an RCV of 30% around the 99th percentile may be more suitable [34,35].

In conclusion, we have demonstrated that the Common Change Criteria (3C) method can yield similarly high specificity estimates for MI and an acute composite cardiovascular outcome which may be used rather than the relative >20% criterion or assay-specific absolute change values for samples that are collected 3 h apart. Additional evaluations and assessments of the 3C method for MI diagnosis in different and contemporary ACS populations with additional cardiac troponin assays are urgently needed to further demonstrate its generalizability.

## Figures and Tables

**Table 1:** Diagnostic parameters for the different analytical change criteria for MI and the composite outcome for the Abbott hs-cTnI assay.

ABBOTT hs-cTnI	MI			Composite Outcome		
Criteria	>20%	>14 ng/L	3C	>20%	>14 ng/L	3C
**Specificity**	66.5% (63.1–69.9%)	98.6% (97.5–99.3%)	93.9% (92.0–95.5%)	65.9% (62.2–69.5%)	99.1% (98.1–99.7%)	95.1% (93.1–96.6%)
**Sensitivity**	59.3% (47.8–70.1%)	66.7% (55.3–76.8%)	53.1% (41.7–64.3%)	42.5% (35.3–49.9%)	31.7% (25.1–38.9%)	30.7% (24.1–37.8%)
**Positive Likelihood Ratio**	1.8 (1.4–2.2)	46.9 (25.6–86.0)	8.7 (6.2–12.3)	1.2 (1.0–1.5)	35.4 (15.5–80.6)	6.2 (4.2–9.2)
**Negative Likelihood Ratio**	0.6 (0.5–0.8)	0.3 (0.2–0.5)	0.5 (0.4–0.6)	0.9 (0.8–1.0)	0.7 (0.6–0.8)	0.7 (0.7–0.8)
**Positive Predictive Value**	15.6% (13.1–18.5%)	83.1% (72.8–90.0%)	47.8% (39.3–56.4%)	25.7% (22.1–29.7%)	90.8% (81.2–95.7%)	63.3% (53.7–72.0%)
**Negative Predictive Value**	94.0% (92.3–95.3%)	96.6% (95.4–97.5%)	95.0% (93.8–96.0%)	80.5% (78.3–82.5%)	83.9% (82.6–85.2%)	83.1% (81.7–84.5%)

Abbreviations: hs-cTnI, high-sensitivity cardiac troponin I; MI, myocardial infarction; 3C, common change criteria.

**Table 2:** Diagnostic parameters for the different analytical change criteria for MI and the composite outcome for the Roche hs-cTnT assay.

ROCHE hs-cTnT	MI			Composite Outcome		
Criteria	>20%	>9 ng/L	3C	>20%	>9 ng/L	3C
**Specificity**	88.1% (85.6–90.3%)	97.0% (95.6–98.1%)	97.2% (95.7–98.2%)	87.9% (85.2–90.3%)	97.8% (96.3–98.7%)	97.5% (96.0–98.5%)
**Sensitivity**	45.7% (34.6–57.1%)	54.3% (42.9–65.4%)	45.7% (34.6–57.1%)	25.8% (19.7–32.7%)	28.0% (21.6–35.0%)	22.6% (16.8–29.3%)
**Positive Likelihood Ratio**	3.8 (2.8–5.2)	18.3 (11.7–28.7)	16.1 (10.0–25.9)	2.1 (1.6–2.9)	12.5 (7.2–21.6)	8.9 (5.2–15.2)
**Negative Likelihood Ratio**	0.6 (0.5–0.8)	0.5 (0.4–0.6)	0.6 (0.5–0.7)	0.8 (0.8–0.9)	0.7 (0.7–0.8)	0.8 (0.7–0.9)
**Positive Predictive Value**	28.7% (22.9–35.3%)	65.7% (55.0–75.0%)	62.7% (51.1–73.0%)	37.2% (30.1–44.9%)	77.6% (66.6–85.7%)	71.2% (59.0–80.9%)
**Negative Predictive Value**	93.9% (92.7–95.0%)	95.3% (94.1–96.3%)	94.5% (93.3–95.4%)	81.0% (79.6–82.3%)	83.0% (81.7–84.2%)	81.9% (80.7–83.0%)

Abbreviations: hs-cTnT, high-sensitivity cardiac troponin T; MI, myocardial infarction; 3C, common change criteria.

**Table 3:** Diagnostic parameters for the different analytical change criteria for MI and the composite outcome for the Ortho cTnI-ES assay.

ORTHO cTnI-ES	MI			Composite Outcome		
Criteria	>20%	>4 ng/L	3C	>20%	>4 ng/L	3C
**Specificity**	42.3% (38.7–45.8%)	71.7% (68.4–74.9%)	95.9% (94.2–97.2%)	38.9% (35.2–42.7%)	71.8% (68.2–75.1%)	97.5% (96.0–98.5%)
**Sensitivity**	60.5% (49.0–71.2%)	79.0% (68.5–87.3%)	56.8% (45.3–67.8%)	46.8% (39.4–54.2%)	50.5% (43.1–57.9%)	29.6% (23.1–36.7%)
**Positive Likelihood Ratio**	1.0 (0.9–1.3)	2.8 (2.4–3.3)	13.7 (9.3–20.3)	0.8 (0.6–0.9)	1.8 (1.5–2.2)	11.6 (6.9–19.6)
**Negative Likelihood Ratio**	0.9 (0.7–1.2)	0.3 (0.2–0.4)	0.5 (0.4–0.6)	1.4 (1.2–1.6)	0.7 (0.6–0.8)	0.7 (0.7–0.8)
**Positive Predictive Value**	9.9% (8.3–11.7%)	22.6% (20.0–25.5%)	59.0% (49.4–68.0%)	17.5% (15.3–20.1%)	33.2% (29.2–37.5%)	76.4% (65.8–84.5%)
**Negative Predictive Value**	91.1% (88.5–93.1%)	97.0% (95.5–98.0%)	95.5% (94.3–96.5%)	72.4% (69.0–75.6%)	83.9% (81.7–85.9%)	83.3% (81.9–84.5%)

Abbreviations: cTnI, cardiac troponin I; MI, myocardial infarction; 3C, common change criteria.

**Table 4:** Diagnostic parameters for the different analytical change criteria for MI and the composite outcome for the Ortho hs-cTnI assay.

ORTHO hs-cTnl	MI			Composite Outcome		
Criteria	>20%	>4 ng/L	3C	>20%	>4 ng/L	3C
**Specificity**	(74.6% 71.3–77.6%)	93.9% (92.0–95.5%)	95.9% (94.2–97.2%)	75.0% (71.6–78.3%)	95.4% (93.5–96.8%)	96.6% (94.9–97.8%)
**Sensitivity**	59.3% (47.8–70.1%)	79.0% (68.5–87.3%)	56.8% (45.3–67.8%)	41.9% (34.8–49.4%)	43.0% (35.8–50.5%)	29.6% (23.1–36.7%)
**Positive Likelihood Ratio**	2.3 (1.9–2.9)	13.0 (9.7–17.5)	13.7 (9.3–20.3)	1.7 (1.4–2.1)	9.3 (6.3–13.6)	8.6 (5.4–13.6)
**Negative Likelihood Ratio**	0.5 (0.4–0.7)	0.2 (0.1–0.3)	0.5 (0.4–0.6)	0.8 (0.7–0.9)	0.6 (0.5–0.7)	0.7 (0.7–0.8)
**Positive Predictive Value**	19.6% (16.4–23.2%)	57.7% (50.2–64.7%)	59.0% (49.4–68.0%)	31.8% (27.4–36.7%)	72.1% (63.8–79.1%)	70.5% (60.2–79.1%)
**Negative Predictive Value**	94.6% (93.1–95.8%)	97.7% (96.6–98.5%)	95.5% (94.3–96.5%)	82.3% (80.3–84.1%)	85.8% (84.1–87.2%)	83.1% (81.8–84.4%)

Abbreviations: hs-cTnI, high-sensitivity cardiac troponin I; MI, myocardial infarction; 3C, common change criteria.

**Table 5:** Concordance between hs-cTnT (Roche) versus different cTnI assays for MI with the different analytical change criteria.

Concordance Table									
	Roche and Abbott hs-cTnI			Roche and Ortho cTnI-ES			Roche and Ortho hs-cTnI		
	3C	Absolute Change	Percent Change	3C	Absolute Change	Percent Change	3C	Absolute Change	Percent Change
**Kappa (95% CI)**	0.671 (0.581–0.760)	0.704 (0.613–0.796)	0.256 (0.194–0.318)	0.659 (0.565–0.754)	0.234 (0.177–0.292)	0.124 (0.083–0.164)	0.659 (0.565–0.754)	0.564 (0.474–0.654)	0.260 (0.191–0.330)
**Number of Observed Agreements (%)**	810 (95%)	819 (96%)	600 (70%)	812 (95%)	621 (73%)	439 (51%)	812 (95%)	785 (92%)	633 (74%)

## Data Availability

The studies were conducted before data-sharing processes were in place, and thus individual data are not available. The data are not publicly available due to privacy.
